# Epitherapy and immune checkpoint blockade: using epigenetic reinvigoration of exhausted and dysfunctional T cells to reimburse immunotherapy response

**DOI:** 10.1186/s12865-020-00353-0

**Published:** 2020-04-21

**Authors:** Isabella McGoverne, Jenny Dunn, Jacob Batham, Wen Juan Tu, Jeremy Chrisp, Sudha Rao

**Affiliations:** 1 Melanie Swan Memorial Translational Centre, Faculty of Science and Technology, University of Canberra, Canberra, Australia; 2 Gene Regulation and Translational Medicine Laboratory, Immunology Department, QIMR Berghofer Medical Research Institute, Brisbane, Australia; 3 EpiAxis Therapeutics, Brisbane, Australia

**Keywords:** Epigenetics, Immunotherapy, Immune system

## Abstract

**Background:**

Cancer cells subvert natural immunosuppression by upregulating the expression of checkpoint proteins and their ligands. For example, tumor cells expressing programmed death-ligand 1 (PD-L1) induce immune cell tolerance to cancers, thereby facilitating tumor progression. The recent clinical success of immunotherapy, particularly checkpoint blockade, represents a significant advance in cancer therapy. However, many cancers develop resistance to immunotherapies, and the underlying mechanisms and how these might be exploited to overcome resistance still need to be determined.

**Methods:**

T cell dysfunction, in part caused by chronic T cell receptor stimulation, diminishes the capacity for durable responses to checkpoint blockade. Furthermore, T cell populations are phenotypically and functionally heterogeneous, resulting in varying responses to checkpoint blockade. Recent molecular studies of T cell heterogeneity have shown that checkpoint blockade on its own does not alter the epigenetic landscape of T cells, despite epigenetic changes governing T cell phenotype.

**Conclusion:**

Here we argue that epigenetic modifiers can be used to prime and sensitize T cells to immunotherapy. Administering epitherapy in conjunction with checkpoint blockade could decrease T cell exhaustion and immunotherapy resistance in many cancer types.

## Background

The excitement surrounding immunotherapy is justified by the positive results seen in both the preclinical and clinical settings. Advances in immunotherapy in the 19th and early twentieth century were minimal, sporadic, and overshadowed by the successes of chemo- and radiotherapy [1, 2]. However, recent immunotherapeutic discoveries such as checkpoint blockade have revolutionized cancer treatment [1, 3]. Instead of attacking the tumor directly, immunotherapies stimulate the immune system to participate in immunosurveillance; that is, recognize, mark, and destroy cancer cells [2]. An immune response against tumors is mounted in multiple stages: recognition of tumor antigens; T cell activation and proliferation; tumor infiltration with T cells; T cell killing activity; and modulation of the natural immunosuppressive response [3].

Natural immunosuppression exists to prevent autoimmunity, but it is also inadvertently detrimental to T cell efficacy. Tumor cells mediate immunosuppression by hijacking inhibitory checkpoint proteins such as programmed death 1 (PD-1), T cell immunoglobulin and mucin domain 3 (TIM-3), lymphocyte activation gene 3 (LAG-3), and cytotoxic T lymphocyte antigen 4 (CTLA-4) expressed at the surface of T lymphocytes [4, 5]. PD-1 and CTLA-4 have so far received the most attention and as such their roles in cancer immunology are now well characterized [6]. CTLA-4 and PD-1 bind specific ligands, CD80 and CD86 and PD-L1 and PD-L2, respectively, to negatively regulate and thus halt effector T cell proliferation, differentiation, and activation [3, 7]. While programmed death-ligand 1 (PD-L1) is conventionally expressed by immune cells including T cells, macrophages, and dendritic cells, tumor cells can also express PD-L1 on their cell surface and, as it confers tumors with a survival advantage.

Tumor cells exploit such immunosuppressive techniques to avoid detection by the immune system, and without immune evasion cancer could not progress from a single tumor cell to metastatic disease [2, 7]. Downstream effects of immune evasion include inhibiting T cell proliferation, inhibiting the production of cell signaling molecules (such as IL-2), and inducing T cell apoptosis [2]. Drug-induced interference of cancer cell ligands such as PD-L1 and regulatory T cell receptors makes it possible to re-instate the anti-tumor effects of T cells [8]. The first monoclonal antibodies that were tested clinically in human cancers targeted CTLA-4 and PD-1 and have now become common therapies in several cancers. Their use, termed checkpoint blockade, marked a breakthrough in cancer immunotherapy [9, 10]. The CTLA-4-blocking antibody ipilimumab entered into clinical trials in 2000 and was approved by the US Food and Drug Administration (FDA) in 2011 [9]. A 2015 study compiled data from 12 ipilimumab clinical trials and showed that ipilimumab treatment resulted in an ~ 20% five-year survival rate in patients with advanced melanoma [11] compared to the historic 10% five-year survival rate [12]. Several monoclonal antibodies have now been approved for cancer therapy by the FDA either as monotherapies or in combination with chemotherapy.

### Resistance to checkpoint blockade is a major limitation of immunotherapy

While checkpoint inhibitors have no doubt been clinically successful, the problem of blockade resistance has emerged. Efforts are now underway to elucidate the mechanisms behind immune resistance to facilitate the development of therapies to overcome it [3]. There are two broad categories of immunotherapy resistance: *primary resistance*, which describes a tumor completely unresponsive to a novel immunotherapy; and *acquired resistance*, which describes tumors that initially respond to immunotherapy but then relapse and progress [3]. A third type of resistance is also sometimes referred to, termed *adaptive resistance*, which refers to initial recognition of the cancer by the immune system followed by the cancer selecting for immune-resistant phenotypes [2]. Given that immunotherapy use is increasing and broadening, so is acquired resistance: 25–33% of metastatic melanoma patients who initially respond to PD-1 checkpoint blockade stop responding and relapse [2].

The multifaceted, multi-step process of tumor immune responses provides many opportunities for tumors to develop immune resistance. For example, primary resistance to anti-CTLA-4 therapy can be caused by the inability of the major histocompatibility complex (MHC) to recognize tumor-associated antigens. Snyder et al. [13] discovered a peptide sequence that, when absent in melanomas, is associated with ipilimumab or tremelimumab resistance. Furthermore, there is a correlation between a high prevalence of somatic mutations (termed ‘high mutational burden’) and probability of response to checkpoint blockade, especially in melanoma [13], non-small cell lung carcinoma (NSCLC) [14], and triple-negative breast cancer (TNBC) [15]. This phenomenon occurs because a high mutational burden tumor expresses more neoantigens; that is, cancer antigens that can be recognized, marked, and attacked by the immune system [16]. PD-L1-negative tumors, for example, are associated with a low mutational burden in melanoma, primary resistance to pembrolizumab, and decreased survival [3, 17]. Snyder et al. [13] also showed that a low mutational load in melanoma leads to primary ipilimumab resistance due to a related lower affinity of tumor antigens to T cell MHC class I molecules.

Primary immunotherapy resistance can also arise from epigenetic modifications, which are heritable but reversible changes in gene expression caused by proteins bound to double helical DNA. Many of these chromatin-remodeling proteins are mutated in human cancers [18], suggesting that cancer cells exploit epigenetic patterns for tumor development and progression [19]. For this reason, the cancer epigenome has become a major therapeutic research focus, paving the way for novel cancer treatments [18, 20]. With regards to immunotherapy, tumor expression of the CXCL9 chemokine is epigenetically silenced in ovarian cancer in mice, inhibiting T cell infiltration into tumors [21]. Peng et al. [21] reversed this repression with epigenetic modifiers, resulting in re-expression of CXCL9 and an increase in T cell trafficking into tumors. Additionally, this epigenetic therapy improved tumor responsiveness to PD-L1 blockade. This is especially exciting, because ovarian cancers usually do not respond well to immunotherapy due to their low mutational burden [16]. As well as low mutational burden, “cold” tumors – which have little immune cell infiltration, such as in pancreatic and prostate cancers – also indicate poor tumor responses to checkpoint blockade [16].

In addition to the major problem of resistance, immunotherapy, like almost all drugs, has side effects. Immune-related adverse events target several systems and organs, and commonly involve the gastrointestinal tract, endocrine glands, and the respiratory, musculoskeletal and integumentary systems. As a result of the global nature of immune-related adverse events, management of these side effects warrants a multidisciplinary approach [22]. For example, the CTLA-4 antibodies ipilimumab and tremelimumab can result in immune-related adverse events that requires administration of corticosteroids and, less frequently lifelong hormone replacement therapy following inflammation of the thyroid, pituitary, and adrenal glands [9].

Finally, while checkpoint inhibition is a promising cancer treatment, many patients fail to respond or maintain a durable response, in part due to a failure to reinvigorate T cells long term or establish immunological memory in T cells [23].

### T cell dysfunction

After administration of immunotherapy, T cells may successfully activate, proliferate, and infiltrate the tumor microenvironment but not perform their killing activity [3]. T cell dysfunction refers to cytotoxic T cells in the tumor microenvironment that have become ineffective or immuno-tolerant, thereby conferring both primary and acquired resistance. T cell dysfunction is in some part induced by chronic T cell receptor stimulation and therefore occurs in disease states such as chronic viral infection and cancer [24, 25]. Prolonged signaling to T cell receptors increases the expression of inhibitory immune checkpoint receptors, which in turn drive T cell dysfunction [24, 26, 27]; persistent PD-1 expression is a typical offender in that regard [9, 24–31]. Moreover, simultaneous expression of inhibitory receptors such as PD-1, CTLA-4, and TIM-3 correlates with increased T cell dysfunction in cancer [24, 28, 29] and tumor progression [27]. As the proportion of T cells that co-express these receptors increases, so does the level of dysfunction [24]. Furthermore, as the number of receptors on tumor-infiltrating lymphocytes increases, activator and effector functions decrease and tumors progress [27].

Although T cell dysfunction was initially described in lymphocytic choriomeningitis virus (LCMV) infected mice, this data laid the framework for understanding T cell dysfunction in cancer. The molecular signature of CD8^+^ cells in chronic viral infection has since been determined [24, 29]. Cytotoxic T cells are the pathogen killing cells of the immune system recognizable by their expression of the cell surface marker CD8. By comparing the gene expression profiles of functional and dysfunctional CD8^+^ cells, Wherry et al. [29] uncovered a phenotypic difference between the two states. Ribosomal subunits responsible for protein production were transcriptionally downregulated in dysfunctional CD8^+^ cells, perhaps explaining the subsequent reduction in cytokine production [24, 29]. Metabolic changes may be a cause of both substantial reduction in cell size and impaired proliferative ability [29]. Dysfunction progresses over time in stages, with IL-2 (a lymphocyte regulator) production diminishing early and IFNγ and TNFα loss occurring later [27]. Furthermore, T cells progress into dysfunction at various times, giving rise to considerable heterogeneity [27].

### T cell exhaustion

Exhausted T cells are a distinct subset of dysfunctional T cells with poor effector function that arise in response to chronic viral infection and cancer [31, 32]. In an acute infection, effector T cells usually give rise to memory T cells but, in chronic infection and cancer, the persistent T cell receptor stimulus gives rise to progressive dysfunction until T cells become exhausted [33]. T cell exhaustion is characterized by the sustained upregulation of checkpoint proteins including PD-1, TIM-3, CTLA-4, and LAG-3 [24, 25, 29, 33]. Exhausted T cell populations are heterogeneous in function and phenotype according to varying expression of checkpoint proteins [25, 34]. Im and colleagues [34] discovered that PD-1 checkpoint blockade in LCMV only causes a proliferative response in a subset of stem-like CD8^+^ cells distinct from CD8^+^ terminal effectors, which differentiate into terminally exhausted CD8^+^ cells. These CD8^+^ cells were positive for CXCR5, the chemokine receptor, intermediate for PD-1, and negative for TIM-3, consistent with other literature [23, 35]. This population has since become known as stem-like or progenitor exhausted T cells, which are distinct from terminally exhausted tumor-infiltrating lymphocytes [25].

Several studies have now shown that checkpoint blockade can at least partially reinvigorate T cell function [33]. For example, while PD-1 blockade in NSCLC patients was successful, only a small proportion of T cell function was restored. When a high proportion of T cells had high PD-1 expression, there was minimal T cell reinvigoration, suggesting that PD-1 expression in tumor-infiltrating lymphocytes may predict responses to PD-1 blockade [27] and that the level of inhibitory receptor expressed by T cells regulates T cell functionality [33]. Conversely, terminally exhausted T cells express high levels of inhibitory receptors such as PD-1 and TIM-3 and do not respond to PD-1 checkpoint blockade in chronic viral infections [34, 35]; they do, however, have superior cytotoxicity but a shorter life span. To address why checkpoint blockade does not universally re-establish T cell effector function, Miller and colleagues [25] examined the features of specific exhausted CD8^+^ cell subsets. They found that dysfunctional T cell populations are heterogeneous and are composed of progenitor or stem-like exhausted T cells as well as terminally exhausted T cells. Furthermore, they found that the exhausted T cell populations in chronic viral infection and tumors have similar protein expression, whereas the exhausted population in fact comprised four major cell types: proliferating cells, effector-like cells, terminally exhausted cells, and progenitor cells.

By examining these differences between progenitor and terminally exhausted T cells, Miller et al. [25] further ascertained the functions of both populations and what facilitates or blocks responses to checkpoint blockade. Progenitor exhausted T cells were found to have superior proliferation potential and be able to differentiate into terminally exhausted cells in vivo, both contributing to their better control of tumor growth than terminally exhausted T cells. Further, PD-1 checkpoint blockade caused proliferation of progenitor exhausted cells and promoted differentiation into terminally exhausted T cells in murine melanomas [25]. PjuiTherefore, PD-1 checkpoint blockade caused temporary reinvigoration of a subset of T cells but not long-term tumor or viral control. Pauken et al. [23] found that PD-1 pathway blockade was not durable over the long term and eventually resulted in T cell “re-exhaustion”. Finally, in addition to altered protein expression in exhausted T cells, transcriptional programs are similarly altered in exhausted T cells [29, 35].

Since chronic activation promotes T cell exhaustion, it remains unclear how checkpoint blockade mediates proliferative responses of tumour-infiltrating T cells. Recently, pre-curser exhausted T cells expressing the transcription factor, TCF7, have been identified as promoters of checkpoint blockade therapy response. Transcriptome profiling of immune cells from melanoma patients identified the transcription factor TCF7 in CD8^+^ T cell subsets associated with positive clinical outcome of checkpoint treated patients [36]. Furthermore, Siddiqui et al. (2019) identified that intratumoral Tcf1^+^PD-1^+^CD8^+^ stem-like T cells are critical for tumour control in response to immunotherapy [37].

### Epigenetics

**Fig. 1:**
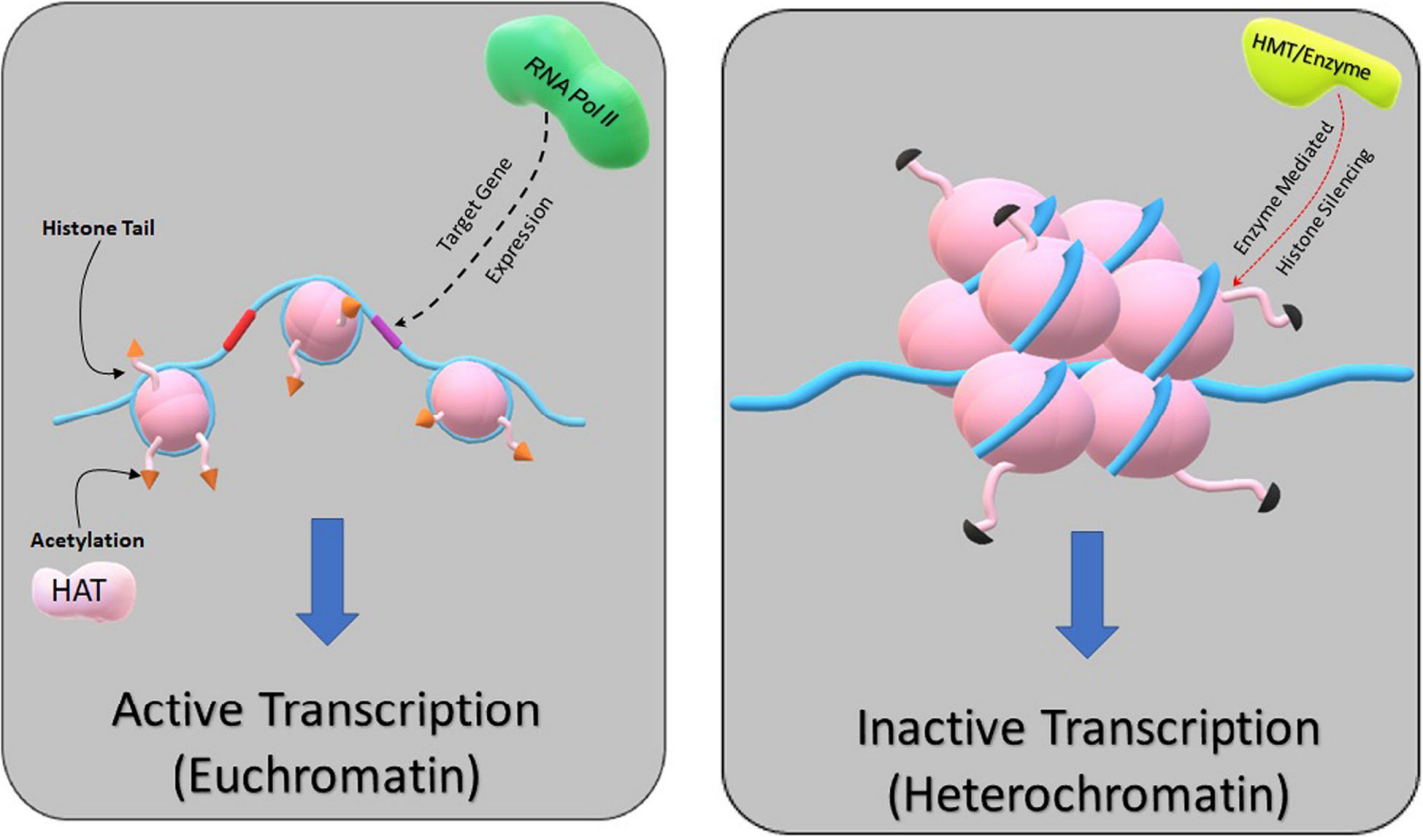
Epigenetic mechanisms such as acetylation of histones via histone acetyl transferase enzymes cause flux between euchromatin and heterochromatin. This results in altered gene expression. It is important to note that not all epigenetic enzymes are characterized as either activators or inactivators of transcription. For example, LSD1 can act as both as co-repressor or co-activator of transcription, dependent on the target residue. Cancer genomes utilize epigenetic mechanisms in tumorigenesis

As noted above, most cancers express epigenetic mutations that contribute to cancer development. Gene expression is regulated by epigenetic mechanisms that control the flux between euchromatin (open chromatin) and heterochromatin (closed chromatin). The main epigenetic mechanisms include post-translational histone modifications and DNA methylation. Post-translational histone modifications are coordinated by epigenetic enzymes that catalyze the addition and removal of functional groups. This mechanism begins with disrupted nucleosome stability, which disturbs the interactions between nucleosomes and DNA, in turn loosening or tightening higher-order chromatin folding and the switching on and off of genes and gene translation [18] (Fig. 1). For example, acetylation of a histone lysine residue neutralizes its basic nature, resulting in an overall euchromatic (open chromatin) effect [38]. Acetyl and other histone marks are covalently added by writer enzymes, and the plasticity of the epigenome is underpinned by the ability of these marks to be removed by eraser enzymes [18].

Epigenetics drugs are pharmaceuticals that target proteins and genes found in the epigenome of the immune system, cancers and other diseases. Epigenetic drugs are divided into two major classes: those that target writers and those that target erasers [8, 39] . These epigenetic drugs, or epitherapies, induce anti-cancer responses by amplifying or reactivating transcription of tumor suppressor genes or cell cycle regulation genes, respectively [20]. This has downstream phenotypic effects on tumor cells including apoptosis, growth inhibition, and inducing cellular differentiation [8]. Whereas chemotherapy and radiotherapy are broad-spectrum cytotoxic treatments with side effects including autoimmune-like attack, epigenetic drugs are more targeted, with some designed to specifically identify and target proteins found only in cancer cells [39]. Several drugs targeting epigenetic witers and erasers and been FDA-approved in cancer treatment (Table 1).

**Table 1:** Epigenetic writers and erasers approved for treatment or in clinical trial in cancer treatment

	Drug name	Commercial name	Class	Company	FDA-approved indication
Epigenetic Writers	Azacitidine	Vidaza	DNMTi	Celgene Corp	AML, CML, MDS
	5-Aza-2′-deoxycytadine	Dacogen	DNMTi	Eisai	AML, CML, MDS
	Tazemetostat	Tazverik	HMTi	Epizyme Inc	Epithelioid Sarcoma*
Epigenetic Erasers	Panobinostat	Farydak	HDACi	Novartis	Multiple Myeloma
	Vorinostat	Zolinza	pan-HDACi	Merk	CTCL
	Belinostat	Beleodaq	pan-HDACi	Spectrum pharmaceuticals	PTCL
	Romidepsin	Istodax	Class I HDACi	Celgene	CTCL/PTCL
	Chidamide	Epidaza	pan-HDACi	Chipscreen Biosciences	PTCL

Abbreviations: DNMTi: DNA methyltransferase inhibitors: HMTi: Histone methyltransferase inhibitor: HDACi: Histone deacteylase inhibitors: AML: Acute myeloid leukemia: CML: MDS: Myeloid dysplastic syndrome: CTCL: Cutaneous T-cell Lymphoma: PTCL: Peripheral T cell lymphoma. * approved in China only

Epigenetic inhibitors are able to reimburse or overcome immune resistance to immunotherapy treatment by upregulation of chemokine expression, antigen processing and presentation machinery, and immune checkpoint molecules. In particular, DNA methyltransferase (DNMT) and histone deaceytlase (HDAC) inhibitors are most well studied classes of epigenetic drugs to date. These inhibitors are able to enhance the expression of several antigen presenting molecules, co-stimulatory molecules, and checkpoint ligands [8]. Other epigenetics enzymes, such as the histone methyltransferase, EZH2, have been shown to regulate the expression of PD-L1 in hepatocellular carcinoma by upregulating H3K27me3 levels on the promoters of CD274 (encoding PD-L1) and IRF1 [40]. Furthermore, BET bromodomain inhibitors have been shown to upregulate PD-L1 and MHC I expression in ovarian and prostate cancer cells [41, 42].

### Epigenetics of T cells

In addition to altered epigenetic states between tumor and normal cells, exhausted T cells are now known to have epigenetic patterns distinct from those of effector and memory T cells [23, 25, 31, 35, 43]. By comparing functional and exhausted T cells in chronic viral infection, Sen et al. [31] established that there was a large difference between their levels of chromatin accessible regions. Chromatin accessible regions were associated with increased gene expression rather than gene repression. In dysfunctional T cells, these regions were adjacent to genes overexpressed in exhausted cells such those encoding PD-1 and TIM-3, reinforcing the idea that checkpoint upregulation in dysfunctional T cells occurs at the epigenetic level and that epigenetic gene regulation programs are specific the various T cell states. Jadhav et al. [43] found two populations of T cells in LCMV mice: exhausted T cells and stem-like PD-1^+^ TCF1^+^ T cells that were epigenetically distinct. Furthermore, the stem-like T cell population was responsible for the T cell proliferative burst following anti-PD-1 therapy.

The efficacy of epitherapies in stimulating immune responses in cancer has been tested. For example, the DNA hypomethylating agent decitabine can increase PD-1 expression silenced by DNA methylation in leukemia, which, in part, makes it more responsive to PD-1 checkpoint blockade [44]. It is now suggested that DNA demethylation evokes genome wide changes, outside the PD-1 locus, which are responsible for changes in responsiveness to anti-PD-1 therapy [45].

CD8^+^ T cell exhaustion and T cell differentiation during exhaustion are regulated by transcription factors, particularly EOMES and T-bet [29, 46]. A study in LCMV mice found expression of genes adjacent to *Eomes* differed in acute versus chronic viral infection; those in acute infection were involved with effector function, whereas those in chronic infection were involved with T cell differentiation and were progressively upregulated. EOMES appears to play different roles in acute infection and T cell dysfunction [35]. PD-1^high^ T cells are known to be associated with exhaustion, whereas PD-1^int^ cells can be reinvigorated by checkpoint blockade. Doering et al. [46] found that T-bet was associated with different genes in PD-1^high^ and PD-1^int^ cells: in PD-1^high^ cells, T-bet-associated genes included those associated with T cell exhaustion such as *LAG3* and *CTLA4*. Therefore, PD-1 expression in heterogeneous T cell populations is driven by gene expression, consistent with the literature showing that T-bet and EOMES play a role in T cell differentiation following tumor antigen recognition.

### Epitherapy and combination therapy

Therefore, many cancer patients have an inherently functional immune system that has been rendered inactive by checkpoint ligand-receptor interactions. As noted above, checkpoint blockade only partially reinvigorates the immune system and provides durable tumor immune responses in only a subset of patients [6]. Furthermore, immunotherapy has not been successful in some particularly aggressive cancers such as triple-negative breast cancer (TNBC). A clinical trial showed that only 18.5% of TNBC (so named for their lack of progesterone, estrogen, and HER-2 receptors) patients with PD-L1^+^ tumors responded to the anti-PD-1 antibody pembrolizumab, and the response rate to avelumab (a PD-L1 monoclonal antibody) in TNBC was only 8.6%. A clinical trial examining the efficacy of combining atezolizumab and the chemotherapy nab-paclitaxel (Abraxane) in TNBC saw a response rate of 39%; however, the duration of response was limited to nine months [47]. Another trial with 902 TNBC patients compared the administration of atezolizumab plus nab-paclitaxel with nab-paclitaxel alone, the former increasing progression-free survival from 5.5 months to 7.2 months [48].

**Fig. 2:**
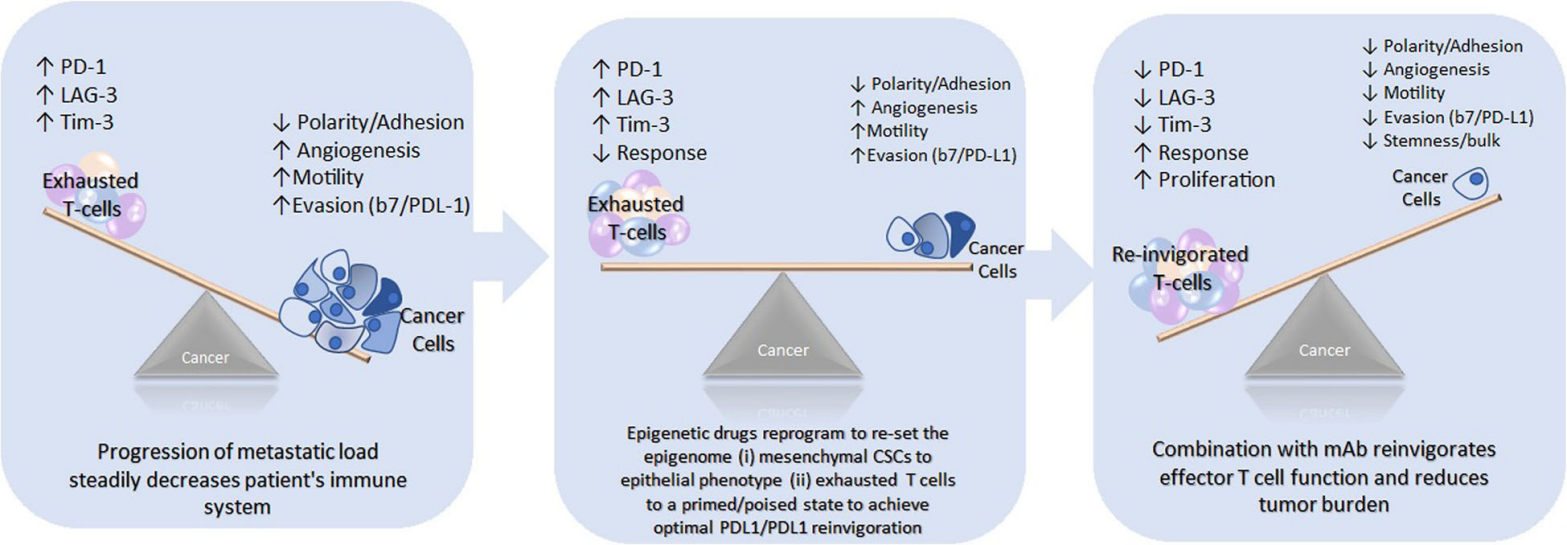
Left: As cancer cells proliferate and tumors progress, T cell receptors become chronically stimulated, lose their effector functions, and become exhausted. Middle: In contrast to immunotherapies, the administration of epitherapies alters the gene expression of T cells. Epitherapy may “level the playing field” between cancer and immune cells by reducing tumor aggression through reprogramming of cancer stem cells from the mesenchymal to the epithelial phenotype. Additionally, epitherapies prime different subsets of exhausted T cells to better respond to immunotherapy. Different classes of epigenetic therapy have distinct transcriptional roles in resetting the epigenome. Right: Once primed by epitherapies and treated with monoclonal antibodies, such as blockade to the PD-1/PD-L1 pathway, T cells have optimal capacity for reinvigoration long term

Alternatively, epitherapy may foster an epigenetic landscape in the immune system and tumor microenvironment that is conducive to long-term immunotherapy responses (Fig. 2). Several research teams including Chiappinelli et al. [20], Jones, Issa and Baylin [18], Ribas and Wolchok [9] and Dunn and Rao [8] believe that the preclinical and clinical data strongly indicate that epitherapy is an excellent primer that could sensitize cancer cells to immunotherapy as well as minimize immunotherapy resistance. Cancer cells acquire both genetic and epigenetic mutations, and both are used to evade immune surveillance [18]. Pauken et al. [23] found that PD-1 blockade alone had little effect on the epigenetic landscape of exhausted T cells, but concluded that combining epigenetic modifying drugs with checkpoint blockade might improve the success of T cell re-invigoration. This is consistent with findings in a recent, comprehensive study by Miller and colleagues which found that PD-1 blockade had little effect on the epigenome of progenitor or terminally exhausted T cells [25]. There are currently several clinical trials underway testing the efficacy of combining epitherapy with other cancer treatments including immunotherapy, chemotherapy, and radiotherapy (Table 2).

**Table 2:** Current clinical trials combining epitherapy with other cancer therapies in various cancer types

Clinical trials identifier	Recruitment status	Phase	Cancer type	Epigenetic drug	Other drug
NCT03812796	Recruiting	2	GI Cancer	Domatinostat (HDACi)	Avelumab
NCT02395627	Active, not recruiting	2	Breast Neoplasms	Vorinostat (HDACi)	Pembrolizumab, Tamoxifen
NCT01928576	Recruiting	2	Non-Small Lung Cancer	Azacitidine (DNMTi), Entinostat (HDACi)	Nivolumab
NCT02512172	Active, not recruiting	1	Colorectal Cancer	Azacitidine, Istodax (HDACi)	Pembrolizumab
NCT02900560	Recruiting	2	Epithelial Ovarian Cancer	Azacitidine	Pembrolizumab
NCT03220477	Recruiting	1	Lung Cancer	Guadecitabine (DNMTi), Mocetinostat (HDACi)	Pembrolizumab
NCT02250326	Active, not recruiting	2	Carcinoma, NSCLC	Azacitidine	Duravalumab, Nab-paclitaxel
NCT01845805	Recruiting	2	Pancreatic Cancer	Azacitidine	Possibly Abraxane or Gemcitabine
NCT02489903	Recruiting	2	Small Cell Carcinoma, NSCLC, Neuroendocrine Tumors, Ovarian Epithelial Cancer	RRx-001	Cisplatin, Etoposide, Carboplatin, Paclitaxel, Nab-Paclitaxel, Pemetrexed
NCT02959437	Active, not recruiting	1/2	NSCLC, CRC, HNSCC, Urothelial Carcinoma, Melanoma	Azacitidine, INCB059872 (LSD1 inhibitor)	Pembrolizumab, Epacadostat, INCB057643
NCT02711956	Active, not recruiting	1/2	Metastatic Castration-Resistant Prostate Cancer	ZEN003694	Enzalutamide
NCT02497404	Active, not recruiting	2	Acute Erythroblastic Leukemia, Myelodysplastic Syndromes	5-Azacytidine	Fludarabine, Melphalan, Alemtuzumab, Radiation
NCT03901469	Recruiting	2	Triple Negative Breast Cancer	ZEN003694	Talazoparib
NCT03179943	Recruiting	2	Urothelial Carcinoma	Guadecitabine (DNMTi)	Atezolizumab
NCT02518958	Active, not recruiting	1	Malignant Solid Tumor, Lymphoma	RRx-001	Nivolumab
NCT03843528	Recruiting	1	Leukemias	Vorinostat, Azacitidine	None
NCT02724202	Active, not recruiting	1	Metastatic Colon Cancer	Curcumin	5-flurorouracil
NCT03505528	Recruiting	1	Metastatic Breast Cancer	Phenelzine Sulfate	Nab-paclitaxel
NCT01627041	Active, not recruiting	2	Acute Adult Leukemia	Decitabine (DNMTi)	Cytarabine and Daunorubicin
NCT03164057	Recruiting	2	AML	Azacitidine or Decitabine	Cytarabine, Daunorubicin, Etoposide, Idarubicin, Fludarabine, Mitoxantrone, Filgrastim, Dexrazoxane, Erwinia asparaginase, Sorafenib
NCT02546986	Active, not recruiting	2	Carcinoma, NSCLC	Azacitidine	Pembrolizumab
NCT03263936	Recruiting	1	Acute Myelogenous Leukemia	Decitabine, Vorinostat	Filgrastim, Fludarabine, Cytarabine
NCT02717884	Recruiting	1/2	AML, Myelodysplastic Syndrome	Tranylcypromine (LSD1 inhibitor)	All-trans Retinoic Acid (Vesanoid), Cytarabine
NCT01534598	Recruiting	1	Neoplasms	5-fluoro-2′-deoxycytidine (FdCyd) (DNMTi)	Tetrahydrouridine (THU)
NCT02951156	Active, not recruiting	3	Diffuse Large B-Cell Lymphoma	Azacitidine	Avelumab, Utomilumab
NCT03417427	Recruiting	2	AML	Decitabine	Ara-C (Cytarabine)
NCT03719989	Not yet recruiting	2	Diffuse Large B Cell Lymphoma, Non-Hodgkin Lymphoma	Azacitidine	Rituximab-GDP
NCT03765229	Recruiting	2	Melanoma	Entinostat	Pembrolizumab
NCT02452970	Active, not recruiting	2	Cholangiocarcinoma	RRx-001	Gemcitabine and Cisplatin
NCT03612739	Not yet recruiting	1	AML	5-Azacytidine	NKR-2
NCT03709550	Not yet recruiting	1/2	Prostate Carcinoma	Decitabine	Enzalutamide
NCT01700569	Recruiting	1	Grade IV Astrocytoma, Glioblastoma	Folinic acid (DNA methylator)	Temozolomide, Radiation
NCT03903458	Recruiting	1	Malignant Melanoma	Tinostamustine	Nivolumab
NCT04022005	Not yet recruiting	2	Lymphoma, Large B-Cell, Diffuse	Chidamide (HDACi)	Rituximab, Gemcitabine, Oxaliplatin
NCT02085408	Active, not recruiting	3	Leukemia	Clofarabine (DNA hypomethylator) with Decitabine	
NCT02842827	Completed	1	AML, Myelodysplastic Syndrome	IMG-7289 (LSD1 inhibitor)	All-trans Retinoic Acid (Vesanoid)
NCT02273102	Active, not recruiting	1	Acute Myelogenous Leukemia	Tranylcypromine (LSD1 inhibitor)	Tretinoin
NCT02712905	Recruiting	1/2	Solid Tumors and Hematologic Malignancy	INCB059872, Azacitidine	All-trans Retinoic Acid, Nivolumab

Abbreviations: *NSCLC* non-small cell lung carcinoma, *HNSCC* head and neck squamous cell carcinoma, *AML* acute myeloid leukemia, *CRC* colorectal cancer, *DNMTi* DNA methyltransferase inhibitor

In fact, several epigenetic inhibitors, such as EZH2 and DNMT inhibitors have been shown to improve the efficacy of immunotherapy treatments such as anti-CTLA-4 and anti-PD1 treatment. For example, Goswami et al. (2018) showed that modulation of EZH2 expression in T cells improves efficacy of anti-CTLA-4 therapy in vivo [49]. Similarly, the DNMT inhibitor decitabine enhanced lymphocyte migration and function and synergized with CTLA-4 blockade in a murine ovarian cancer model [50]. Furthermore treatment with decitabine was shown to enhance the effect of PD-1 blockade in colorectal cancer by re-modulating the tumor microenvironment [51]. Improved responses have also been observed with other classes of epigenetic drugs. For example, targeted inhibition of the PD-1/PD-L1 axis by combining anti-PD-1 antibodies and the BETi JQ1 caused synergistic responses in mice bearing Myc-driven lymphomas [52]. These studies provide a strong rationale for a combination of epigenetic and immunotherapy treatment in cancer therapy.

## Conclusion and future directions

Reinvigorating an ineffective immune system has become a cornerstone of cancer therapy. While monoclonal antibodies are showing great promise in promoting immunogenicity, the clinical reality is that immune reinvigoration is thwarted by primary and acquired resistance. Cancer epigenetics is an established field of significant interest in terms of both its contribution to carcinogenesis and gene expression alterations in the cancer patient’s immune system – and the complex interplay between the two. Combinations of epitherapy with established therapies have been shown to slow cancer progression at the clinical trial level, with epitherapy used to selectively reduce or re-establish the expression of genes that promote tumorigenesis and immunogenicity, respectively. Future studies in the field of epigenetics, T cell exhaustion, and cancer include developing new therapies, including combinations of therapies, for cancers unresponsive or that have low responsiveness to immunotherapy, such as prostate cancer. Furthermore, while the molecular biology of T cell exhaustion has been established, a lot of the relevant research has been in virus models and specific research into exhaustion in cancer models is warranted. Finally, many epigenetic proteins and their downstream cellular effects remain poorly characterized, even though they may have implications in cancer and T cell exhaustion. Identification of these mechanisms will facilitate further development of targeted epigenetic drugs.

## Data Availability

Not applicable.

## Abbreviations

AML: Acute myeloid leukemia
CLC: Chronic lymphocytic leukemia;
CRC: Colorectal cancer
CTLA-4: Cytotoxic T lymphocyte antigen 4
CXCL9: CXC motif ligand 9
DNMTi: DNA methyltransferase inhibitor
FDA: US Food and Drug Administration
HNSCC: Head and neck squamous cell carcinoma
IL-2: Interleukin-2
LAG-3: Lymphocyte activation gene 3
LCMV: Lymphocytic choriomeningitis virus
mAb: Monoclonal antibody
MHC: Major histocompatibility complex
NSCLC: Non-small cell lung carcinoma
PD-1: Programmed death 1
TCF1: T cell factor 1
TIM-3: T cell immunoglobulin and mucin domain 3
TNBC: Triple-negative breast cancer
